# An *in-vivo* Intraoral Defect Model for Assessing the Use of P_11_-4 Self-Assembling Peptide in Periodontal Regeneration

**DOI:** 10.3389/fbioe.2020.559494

**Published:** 2020-09-23

**Authors:** Basmah El-Sayed, Robert Philip Wynn Davies, Rehab R. El-Zehery, Fatma Mohamed Ibrahim, Mohammed E. Grawish, Jennifer Kirkham, Reem El-Gendy

**Affiliations:** ^1^Division of Oral Biology, School of Dentistry, University of Leeds, Leeds, United Kingdom; ^2^Department of Oral Biology, Faculty of Dentistry, Mansoura University, Mansoura, Egypt; ^3^Department of Oral Biology, Faculty of Oral and Dental Medicine, Delta University for Science and Technology, Mansoura, Egypt; ^4^Department of Oral Pathology, Faculty of Dentistry, Suez Canal University, Ismailia, Egypt

**Keywords:** regenerative medicine, biomaterial(s), periodontal disease(s)/periodontitis, nanotechnology, histochemistry

## Abstract

Periodontal disease is one of the most common diseases worldwide. It has a significant impact on oral health and subsequently the individual’s quality of life. However, optimal regeneration of periodontal tissues, using current treatments, has yet to be achieved. Peptide self-assembly has provided a step-change in nanobiotechnology and regenerative medicine fields. Our aim was to investigate the effects of a self-assembling peptide (SAP; P_11_-4) on periodontal regeneration in a preclinical model. Twenty-six bilateral maxillary critical-sized periodontal defects were created surgically in 13 rats. Defects on one side of the mouth were filled with P_11_-4 hydrogel; the contra-lateral defect was untreated (control). Rats were sacrificed immediately post-surgery (time 0) and after 2 and 4 weeks. Retrieved maxillae were processed for histological, immunohistochemical, and histomorphometric assessments. The results of histological analysis showed greater organization of periodontal fibers in defects treated with P_11_-4, at both time points, when compared to untreated defects. Histomorphometry showed that treated defects had both a significant increase in functional periodontal ligament length and a reduction in epithelial down growth after 4 weeks. At 2 weeks, treated defects showed a significant increase in expression of osteocalcin and osteoprotegerin as judged by immunohistochemistry. Also, a significantly higher osteoprotegerin/RANKL ratio was shown in treated defects. In conclusion, the results demonstrated enhanced regeneration of periodontal tissues when SAP P_11_-4 was used to fill periodontal defects in rats. The findings of this study suggest that SAP P_11_-4 is a promising novel candidate for periodontal regenerative therapy. Further investigations are required for optimization before clinical use.

## Introduction

Periodontal disease (PD) adversely affects oral function and esthetics and is a major cause of tooth loss in older adults (Petersen and Ogawa, 2012). In addition, untreated chronic inflammatory PD has been linked to systemic diseases, such as cardiovascular disease (Humphrey et al., 2008). Successful management of PD is, therefore, of central importance not only to oral health but to health *per se*.

The complexity of the three dimensional architecture and tissue composition of the periodontium pose challenges to healing and restoration of full function following destruction by PD (Ivanovski et al., 2014). Conventional treatment strategies halt the progression of disease but may not produce full regeneration of periodontium (Susin et al., 2015). Contemporary surgical treatment enhances periodontal regeneration (PR) through the use of graft materials (Trombelli et al., 2002), growth factors (Kitamura et al., 2016), root surface conditioning (Karam et al., 2016), and guided tissue regeneration (Reynolds et al., 2015) but with sometimes undesirable or unpredictable results (Susin et al., 2015). Alloplastic biomaterials such as ceramics and polymers can overcome these limitations but optimal periodontal reconstruction has yet to be achieved (Lee et al., 2012).

Regenerative therapies are continuing to be developed as potential solutions to intractable clinical problems. A recent review by the American Academy of Periodontology (Lin et al., 2015) concluded that ongoing research in regenerative medicine has led to the development of novel treatment approaches and technologies for PD, including the use of nanomaterials, to improve treatment outcomes (Lin et al., 2015).

There has been a growing interest in self-assembling peptides (SAPs) as nanomaterials for use in regenerative medicine, including as candidates for periodontal regenerative therapy, due to their unique properties (Takeuchi et al., 2016). Zhang and colleagues led the way in developing this technology for use in regenerative therapies (Zhang, 2002).

Self-assembling peptides used in regenerative medicine offer the advantages of being biocompatible, biodegradable and, where required, bioactive. Peptide self-assembly into macromolecular structures mimics natural biological processes (e.g., collagen fibrillogenesis in bone and amelogenin self-assembly in developing enamel) (Palmer et al., 2008), generating three dimensional hydrogel scaffolds with properties similar to extracellular matrices (ECMs). These hydrogels have shown to support cell attachment, proliferation and differentiation, in addition to initiating biomineralization and promoting wound healing (Holmes et al., 2000; Webber et al., 2010).

Self-assembling peptide P_11_-4 was developed as a biomimetic material reflecting the properties of ECMs in mineralized tissues (Kirkham et al., 2007; Kyle et al., 2009; Brunton et al., 2013) and is currently used clinically in the treatment of early caries of enamel. P_11_-4 is a promising candidate in regenerative therapies (Schmidlin et al., 2016) but its full potential has yet to be explored. We hypothesized that P_11_-4 would promote PR based upon its known biocompatibility (Saha et al., 2019) and ability to repair mineralized tissues (Jablonski-Momeni and Heinzel-Gutenbrunner, 2014). Our aim was to investigate the use of P_11_-4 hydrogels on periodontal ligament (PDL) and alveolar bone (AB) regeneration in surgically created periodontal defects in rats.

## Materials and Methods

### Rheology of P_11_-4 as a Function of Concentration

All rheological measurements were carried out on an Anton Paar MCR 302 (Leeds School of Dentistry). Rheo Compas s1.20 by Anton Paar was used to control and export the raw data. A parallel plate geometry with a diameter of 50 mm and a gap of 0.1 mm was used. All tests were run at 37°C with a solvent trap to prevent the evaporation of the aqueous medium. Samples of concentrations 15, 30, and 60 mg/ml were prepared by weighing out the required amount of lyophilized P_11_-4 dissolving in PBS and pH adjusting with aliquots of HCl, following this the gels were then drawn up into a hypodermic syringe. All gels were left to equilibrate for 48 h, after which they were dispensed through the syringe onto the geometries for immediate testing. P_11_-4 at concentrations of 15, 30, and 60 mg/ml were probed with an amplitude sweep in a controlled shear strain state from 0.01 to 100% at 1 and 20 HZ, this determined the linear viscoelastic region (LVER), in which a strain value that lay within this region was chosen. The dynamic modulus was then probed via a frequency sweep for each sample, at the strain value that was established from the amplitude sweep.

### Preparation of SAP P_11_-4

Freeze-dried P_11_-4 (CH_3_COOQQRFEWEFEQQNH_2_) was obtained from Credentis AG (Zurich) with a purity of 98% by HPLC and sterilized using gamma irradiation. Sterile SAP gels were prepared in PBS at least 48 h before surgery at a concentration of 60 mg/ml (pH 7.4). The prepared peptide solutions were drawn up into hypodermic syringe barrels and were allowed to form gels in the syringes over a minimum of 48 h to complete the self-assembly process. The SAPs were then delivered directly from the syringes to the defects on the day of surgery.

### Animals

Thirteen male, pathogen free, Sprague Dawley rats each weighing 150–200 g were used in this study. All experimental procedures were performed according to a protocol approved by the Ethical Committee of the Faculty of Dentistry, Mansoura University, Egypt. The rats were kept under conditions of controlled temperature, humidity and with a 12:12 h light-dark cycle. Animals received standard rat chow and water *ad libitum* unless otherwise stated below.

### Surgical Protocol and Study Design

**FIGURE 1:**
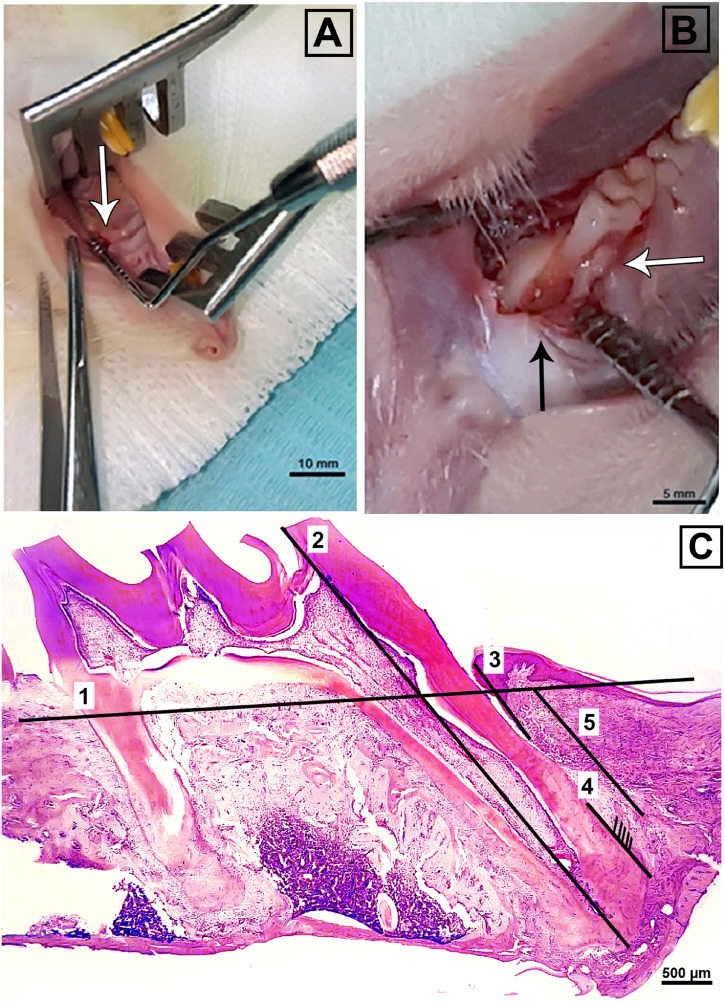
**(A)** Showing magnified view of 3 mm crestal incision, mesial to the maxillary first molar (white arrow) and **(B)** critical-sized periodontal surgical defect (2 mm × 2 mm × 1.7 mm) (black arrow) adjacent to the mesial root of a maxillary first molar (white arrow). **(C)** Illustration of histomorphometric analysis of H&E stained sections. The numbers from one to five indicate the reference level of intact alveolar bone, the length of the first molar mesially from apex to cusp, the length of the epithelium from the free gingival margin to the most apical extent, the length of functional ligament, and length of bone gap till most coronal extent of new bone, respectively. **(A)**; bar = 10 mm, **(B)**; bar = 5 mm, **(C)**; bar = 500 μm.

The study design is illustrated in Supplementary Appendix Table S1. All surgeries were carried out under aseptic conditions at the University of Mansoura. Animals were anesthetized by intraperitoneal injection using ketamine as described previously (Padial-Molina et al., 2012). Surgical bilateral maxillary periodontal defects were created in each hemi-maxilla, just mesial to the first maxillary molar (Bizenjima et al., 2015). Briefly, a 3 mm sagittal surgical incision was made on the crest of the alveolar ridge using a size #11 surgical blade. Buccal and palatal flaps were raised to expose the mesial root of the maxillary first molar. Bilateral critical-sized surgical defects (2 mm × 2 mm × 1.7 mm) were created in the AB using a round bur (1 mm diameter) with a long shank accompanied by cooling with physiological saline solution. A small, sharp curette was used to perform curettage and root planning of the mesial root surfaces to ensure removal of any residual PDL (Figure 1). Defects were then irrigated with physiological saline to flush out any debris. One defect/animal was filled with P_11_-4 hydrogel from a syringe; the contra-lateral defect remained empty. To close the wound, buccal and palatal gingival edges were approximated and stabilized with cyanoacrylate glue (Histoacryl^®^ B. Braun Surgical, S.A. Carretera de Terrassa, 121, 08191 Rubi, Spain). After recovery from anesthesia, Ketoprofen (5 mg/kg) was provided as analgesic for 3 consecutive days. Ampicillin, 268 mg/L, added to a 5% dextrose solution was given via the drinking water as antibiotic treatment (Padial-Molina et al., 2012). Dextrose (5%) was added to the drinking water for a total of 7 days post-surgery to augment nutrition following the intra-oral surgery. One rat was euthanized on the day of surgery (Day 0), six rats were euthanized 2 weeks post operatively while the remaining rats (*n* = 6) were euthanized after 4 weeks. The maxillae of both groups were harvested for histology processing.

### Histological Analysis of Retrieved Defects

Once harvested, all specimens were fixed for 48 h in 10% neutral buffered formalin. Each maxilla was divided into two halves, rinsed with distilled water and then transferred to 10% neutral buffered EDTA for decalcification. EDTA solution was changed daily for approximately 4 weeks. The decalcified hemi-maxillae were then processed and wax embedded for transfer to Leeds. Serial sections (7 μm thickness) were obtained from the central portion of the root and every tenth section was stained with hematoxylin and eosin (H&E) with adjacent slides used for immunohistochemistry (see below). Stained sections were viewed in the Olympus BX51 bright field/fluorescence microscope and images captured with a Nikon Ds-Fi1 digital camera using Nikon NIS Elements imaging software (v3.1) for histomorphometric analysis as described below.

### Histomorphometric Analysis

Hematoxylin and eosin stained sections of periodontium including the defects, located mesial to the mesial root of the maxillary first molar, were assessed for newly formed bone, newly formed functional PDL (defined as described below) and junctional epithelial down-growth. Three sections per hemi-maxilla per animal were subjected to analysis. Thus, a total of 72 H&E stained sections (18 sections per group) were evaluated. The quantitative data were normalized to tooth length in order to compensate for any differences in cutting angulation between samples (Figure 1). The following parameters were assessed by ImageJ digital image analysis (Oortgiesen et al., 2013; Yan et al., 2015) then subjected to statistical analysis:

#### Relative Alveolar Bone Height

A line was drawn at the level of the furcation height as a reference to normal bone height. A line representing the length of the molar was measured from the apex of the mesial root to the cusp. The line parallel to that of the root and extending from the reference line to the level of regenerated bone surface, representing alveolar bone gap, was measured. Relative alveolar bone height was determined by dividing the mesial molar length by the length of bone gap. A higher score indicates superior height of newly formed bone.

#### Relative Epithelial Down-Growth

The length of the epithelium was measured from the gingival margin to the most apical extent of junctional epithelium. Epithelial down-growth was calculated via dividing epithelial length by the length of the mesial molar. A higher score indicates greater epithelial down-growth.

#### Relative Functional Ligament Length

Collagen fibers were included in histomorphometry when the main fibers formed an angle >60° to the long axis of the root (functional PDL). The length of functional ligament was divided by the length of the molar to obtain the relative functional ligament length (Oortgiesen et al., 2013; Yan et al., 2015).

### Immunohistochemical Analysis

Paraffin sections were de-waxed in xylene and processed for immunohistochemical evaluation of expression of proliferating cell nuclear antigen (PCNA), osteocalcin (OCN), collagen type I (COL I), osteoprotegerin (OPG), and receptor activator of nuclear factor kappa-B ligand (RANKL). Supplementary Appendix Table S2 shows the antibodies used in this study, along with their optimized dilutions. Sodium citrate was used for antigen retrieval and immunohistochemical staining performed according to the manufacturer’s instructions for each second antibody used. A total of 360 sections were assessed (72 sections per antibody). For each antibody, three sections per hemi-maxilla per animal were analyzed (18 sections per group). Serial sectioning of the specimens enabled the evaluation of three sections per antibody: one section from the surface, one from the central portion and one from the deep part of the defect area. Images were acquired in the Olympus BX51 bright field/fluorescence microscope at a fixed magnification (×200) and field [connective tissue (CT) adjacent to the root and immediately above the newly formed AB] as described previously. Digital image analysis was used to quantify the proportion of PCNA positive cells. The % area of positive expression was determined for OCN, OPG, and RANKL and the OPG/RANKL ratio calculated (Tan et al., 2009; Peng et al., 2013; Ross, 2014). Observations for COL I connective tissue fiber organization were recorded but slides stained for COLI were not subjected to quantitative analysis given the predominance of type I collagen in all of our samples.

### Statistical Analysis

Mead’s resource equation was used to determine appropriate sample size for the animal experiment (Chen et al., 2016). The data collected from histological and immunohistochemical analyses was analyzed using SPSS v17. Results were expressed as means ± SD. The distribution of all variables was examined for normality using Shapiro-Wilk normality testing. Quantitative analysis parameters were compared using independent samples *T*-test and the significance level was set at *p* < 0.05.

## Results

### Characterization of P_11_-4 Hydrogel

**FIGURE 2:**
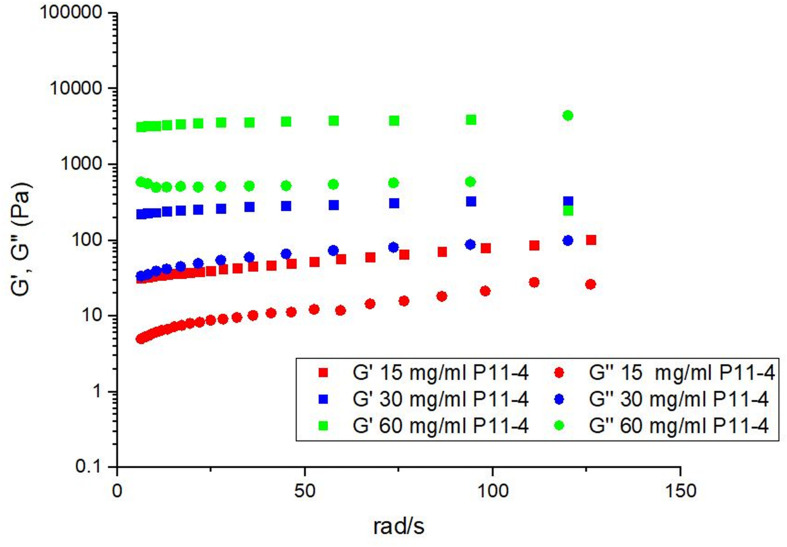
The effect of varying the concentration on the mechanical properties of P_11_-4 showing that upon increasing concentration the stiffness of the gel increases.

In order to determine the optimum peptide concentration of P_11_-4 to administer it was hypothesized that the material must remain in a gel like state as opposed to a solid like ‘paste’ or a weak ‘viscoelastic fluid.’ The material properties were therefore tested to optimize the gel stiffness. In all cases the storage modiolus, G’, corresponding to the elastic component is greater than the loss modiolus, G″, corresponding to the viscous component indicating gel like behavior. Figure 2 shows that both G′ and G″ increase as a function of concentration, indicating that the stiffness of the gels increases as a function of concentration. In the highest concentration we have retained the desired material properties but increased the stiffness by orders of magnitude when compared with the lowest concentration. As the material intervention must remain *in situ* despite mechanical forces applied through normal mastication it was concluded that the most appropriate concentration to take into our animal model was the stiffest gel concentration (60 mg/ml).

### Histological Observations

**FIGURE 3:**
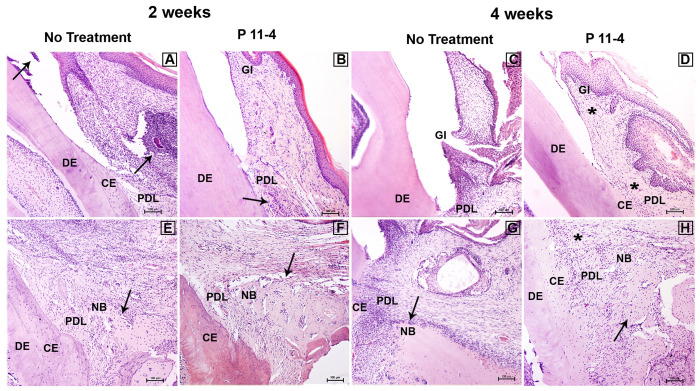
Representative images showing histopathological overview after 2 and 4 weeks of healing (H&E staining) in untreated and P_11_-4-treated defects. Upper panel **(A–D)** shows the gingival region of the defects while lower panel shows the apical region of the defects **(E–H)**. At 2 weeks the untreated group showed evidence of inflammatory cell infiltrate in the gingival connective tissue and gingival sulcus (arrows) **(A)** while the P_11_-4-treated group appeared to show healthier gingival healing with connective tissue and periodontal ligament demonstrating better organization of fibers and cells (arrow) **(B)**. Gingiva and periodontal ligament at 4 weeks also showed enhanced structure in the P_11_-4-treated group (asterisks) **(D,H)**. Newly formed bone structure and trabecular organization appeared to be superior in P_11_-4-treated groups at 2 and 4 weeks (arrows) **(E–H)**. Gingiva (GI), dentin (DE), cementum (CE), periodontal ligament (PDL), and new bone (NB). **(A–H)** ×100; bar = 100 μm.

Both P_11_-4-treated and untreated defects retrieved after 2 and 4 weeks post-surgery showed evidence of newly formed CT, compared with Day 0 defects but the histological appearance/organization of these tissues appeared superior after treatment with P_11_-4 compared to the untreated defects with respect to fiber orientation and bone architecture (Figure 3).

P_11_-4-treated defects showed high condensation of newly formed blood capillaries directly adjacent to AB at 2 weeks, suggesting enhanced angiogenesis, which is potentially beneficial for tissue regeneration and wound healing. In addition, newly formed gingivae displayed highly organized fibers and oriented fibroblasts at both time points after P_11_-4 treatment. Superior orientation of newly formed oblique PDL fibers was also evident. The amount of new bone in P_11_-4-treated defects was not obviously different to that in untreated defects but any new bone was well-constructed with improved trabecular structure at 2 and 4 weeks in the P_11_-4-treated defects. On the other hand, untreated defects showed far less organized newly formed gingiva, PDL fibers and bone (Figure 3).

Finally, inflammatory cell infiltrates in the gingival sulcus and gingival CTs were seen in untreated but not P_11_-4-treated defects after 2 weeks (Figure 3).

### Histomorphometric Analysis of H&E Sections

There were no significant differences in relative epithelial down-growth and relative functional PDL length between P_11_-4-treated and untreated defects at 2 weeks post-surgery. However, relative epithelial down-growth significantly decreased while relative functional PDL length increased after P_11_-4 treatment compared with untreated defects after 4 weeks (*p* < 0.05).

There were no significant differences in AB height between P_11_-4-treated and untreated defects at either time point (Table 1).

**TABLE 1:** Showing histomorphometric analysis results and quantitative analysis of immunohistochemical stains.

		2 weeks’ time point		4 weeks’ time point	
	Data represents	No treatment	SAP P_11_-4	No treatment	SAP P_11_-4
Relative functional ligament length		0.3 ± 0.04	0.29 ± 0.13	0.08 ± 0.07	0.42 ± 0.04*
Relative epithelial down-growth	Measurements in μm	0.15 ± 0.01	0.146 ± 0.01	0.14 ± 0.01	0.09 ± 0.01*
Relative alveolar bone height		3.80 ± 1.54	2.57 ± 0.33	4.13 ± 0.86	5.33 ± 0.28

PCNA	Mean % of positive cells count/total cells count	19.7 ± 0.7	21.2 ± 2.8	21.2 ± 0.4	22.3 ± 3.1
	
OCN	Positive mean % area	14.0 ± 0.9	23.5 ± 2.1*	14.9 ± 1.5	17.4 ± 4.0
OPG		24.7 ± 3.2	31.4 ± 2.4*	18.1 ± 1.4	26.9 ± 1.8*
RANKL		27.9 ± 2.5	31.2 ± 3.3	23.8 ± 2.0	29.9 ± 5.5
OPG/RANKL		0.9 ± 0.04	1.0 ± 0.04*	0.8 ± 0.03	0.9 ± 0.11*

Data shown as mean ± SD (median 95% confidence interval) calculated by independent samples T-test with Levene’s test for equality of variances (*p* < 0.05). *Significant values of P_11_-4 when compared with untreated group at the same time point.

### Immunohistochemical Observations and Analysis

**FIGURE 4:**
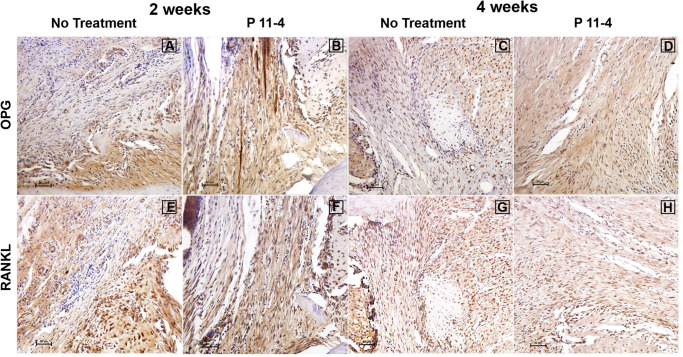
Representative photomicrographs of immunohistochemical staining for osteoprotegerin (OPG) **(A–D)** and receptor activator of nuclear factor kappa-B ligand (RANKL) **(E–H)** in untreated and P_11_-4-treated defects. Connective tissue areas adjacent to the root and immediately above the newly formed bone are shown (original magnification; ×200; bar = 50 μm). OPG expression appeared to be higher in P_11_-4-treated defects when compared with the untreated defects at 2 and 4 weeks **(A–D)**, while RANKL expression seemed comparable in untreated and P_11_-4-treated groups at both time points **(E–H)**. See Table 1 for quantitative values of mean % area for OPG and RANKL, in addition to the mean OPG/RANKL ratios.

Greater levels of OPG expression were noted in P_11_-4-treated defects at 2 and 4 weeks post-surgery, while RANKL expression appeared comparable in both untreated and P_11_-4-treated groups at both time points (Figure 4). Quantitative analysis showed that compared with untreated defects, OPG expression was significantly higher in P_11_-4-treated defects at both 2 and 4 weeks (*p* < 0.05). Moreover, the mean OPG/RANKL ratio was significantly higher in the P_11_-4-treated group at both 2 and 4 weeks suggesting greater potential for osteogenesis. RANKL expression showed no significant differences at either time point (Table 1).

**FIGURE 5:**
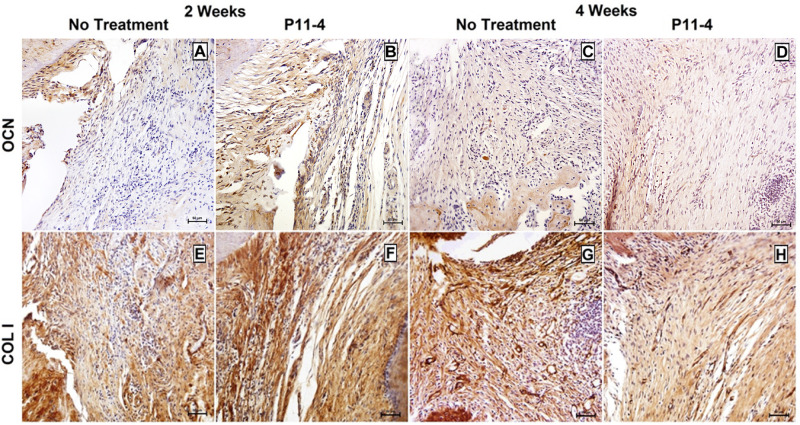
Representative photomicrographs of immunohistochemical staining for osteocalcin (OCN) **(A–D)** and collagen type I (COL I) **(E–H)** in untreated and P_11_-4-treated defects. Connective tissue areas adjacent to the root and immediately above the newly formed bone are shown (original magnification; ×200; bar = 50 μm). Positive OCN expression seemed to be higher in the P_11_-4-treated groups when compared to the untreated groups at 2 **(A,B)** and 4 **(C,D)** weeks post-surgery. Newly formed COL I fibers appeared to be more developed and more regularly arranged in P_11_-4-treated groups at 2 **(E,F)** and 4 **(G,H)** weeks post-surgery.

Positive OCN expression appeared to be higher in the P_11_-4-treated groups when compared to the untreated groups at 2 and 4 weeks (Figure 5). This was confirmed using quantitative analysis at the earlier time point (*p* < 0.05) but OCN expression in the P_11_-4-treated defects compared with untreated defects was not significantly different after 4 weeks (Table 1).

Collagen type-I expression was seen in our area of interest, adjacent to the root and just coronal to the newly formed bone, in both treated and untreated defects. Fibers were, however, more highly organized and aggregated into uniform bundles of similar orientation in the P_11_-4-treated defects after 2 and 4 weeks when compared with untreated defects. Furthermore, comparing the P_11_-4-treated defects after 4 weeks with those after 2 weeks showed a continued improvement in CT architecture, as the fibers displayed a tighter arrangement and higher level of organization, as opposed to the untreated defects which showed limited organization of CT (Figure 5).

**FIGURE 6:**
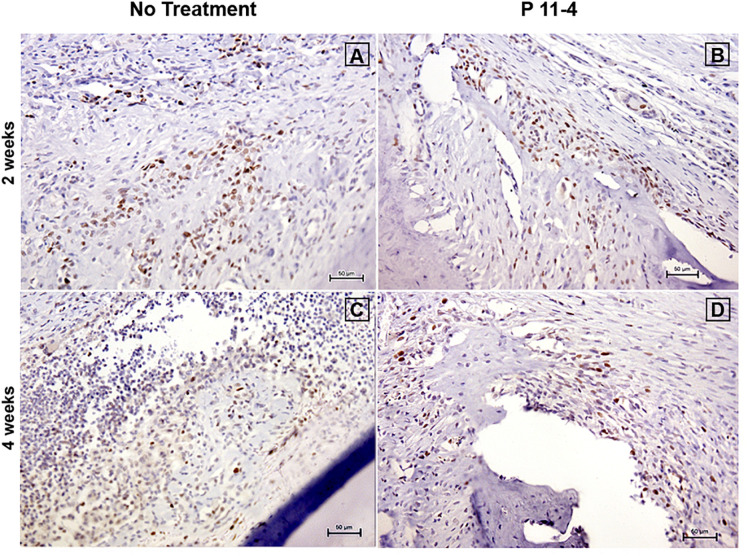
Representative photomicrographs of immunohistochemical staining for proliferating cell nuclear antigen (PCNA) at 2 **(A,B)** and 4 **(C,D)** weeks post-surgery in untreated and P_11_-4-treated defects. Connective tissue areas adjacent to the root and immediately above the newly formed bone are shown (original magnification; ×200; bar = 50 μm). The number of PCNA positive cells in both groups appeared comparable at both time periods.

The proportion of PCNA positive cells in the designated fields appeared comparable when evaluating both groups at 2 and 4 weeks post-surgery (Figure 6). Statistical analysis revealed that there were no significant differences in the proportion of PCNA positive cells between P_11_-4-treated and untreated defects whether at 2 or 4 weeks (Table 1).

## Discussion

The present study was conducted to evaluate the effects of SAP P_11_-4 on regeneration of PDL and AB. SAP P_11_-4 hydrogels were developed specifically for CT regeneration (Aggeli et al., 2003) and are available commercially for treatment of early carious lesions. To the best of our knowledge the current work represents one of the first *in-vivo* studies to evaluate the possible application of P_11_-4 in the regeneration of periodontal tissues in a critical sized defect.

Although P_11_-4 was developed to self-assemble with a charged based trigger mechanism its assembly is still inherently concentration dependent. Therefore, it was hypothesized and shown here that increasing the concentration would yield a stiffer gel. P_11_-4 has shown to be a successful material in treating caries lesions at 10 mg/ml (Brunton et al., 2013) and more recently Saha and colleagues have demonstrated that at 30 mg/ml P_11_-4 is a suitable scaffold for the regeneration of bone in a cranial defect (Saha et al., 2019). Both applications are in areas whereby the mechanical forces on the scaffold its-self are shielded. In this study, however, the regenerative site is known to be under significant force during mastication and to a lesser extent from the applied forces from secreted crevicular fluid in the cavity. It was prudent to develop a formulation that would allow for the SAP matrix to be retained under these forces therefore a concentration with the highest possible elastic and viscoelastic modulus (60 mg/ml) was used to prevent seepage from the treated site.

The results of our study suggested that P_11_-4 enhanced regeneration of periodontal tissues over a period of 4 weeks following surgical induction of critical-sized surgically induced defects. No evidence of local adverse reactions were seen in association with P_11_-4, indicating good biocompatibility as shown previously *in vitro* (Kyle et al., 2012) and more recently *in vivo* (Saha et al., 2019).

The use of SAP hydrogels in tissue engineering in general, and regeneration of the oro-dento–alveolar complex in particular, has shown promising results to date (Pugliese and Gelain, 2017). The fact that P_11_-4 forms hydrogels proved to be beneficial to its application as it allowed the scaffold to be injected into the periodontal defects. Injectability is considered one of the favorable attributes of scaffolds used in tissue regeneration applications (Chang et al., 2017).

Defects treated with P_11_-4 demonstrated evidence of highly organized regenerated gingiva and PDL fibers, especially at the 4 weeks’ post-surgery time point as opposed to untreated controls which demonstrated filling of the defects with CT of limited organization, leading to statistically significant reduction of epithelial down-growth and increase in the mean relative length of functional PDL after 4 weeks. Furthermore, the CT in P_11_-4-treated defects appeared healthier, with a high condensation of newly formed blood capillaries which promote wound healing (Guo and DiPietro, 2010). Our results are consistent with the findings of others using different SAPs, who concluded that SAP nanofiber scaffolds accelerate wound healing and re-epithelialization (Schneider et al., 2008; Meng et al., 2009; Yoshida et al., 2019).

The noticeable impact of P_11_-4 on gingival and PDL regeneration may be related to the findings of Kyle et al. (2012) who demonstrated that primary human dermal fibroblasts infiltrated P_11_-4 hydrogels and aligned at the periphery. Cell numbers within the hydrogel increased over a period of 4 weeks similar to collagen scaffolds and the cells appeared to use the P_11_-4 fibrillar network as a scaffold, similar to ECM proteins, during the first 14 days of incubation. After this, the P_11_-4 hydrogels started to degrade and were replaced by newly formed ECM secreted from the cells (Kyle et al., 2012). The orientation of collagen fibers and gingival fibroblasts seen in association with P_11_-4 treatment in our study is of importance to functionality of the PDL and may reflect the responsiveness of SAP gels to prevalent shear forces (Scanlon et al., 2009).

Despite the fact that P_11_-4 hydrogels failed to produce a significant increase in AB height at 4 weeks, the architectural quality of regenerated bone was improved, with well-defined trabecular structure seen in the P_11_-4 defects. The lack of significant AB height is possibly due to the deficiencies in the material properties (i.e., gel stiffness) of the peptide coupled with the possible degradation of the peptide *in situ*, it is entirely possible that the peptides persistence in the wound site is relatively short. The study presented here did not look at the degradation profile of the peptide, any future larger studies would examine the peptides presence as a function of time. None the less our data indicates a positive effect with the use of P_11_-4.

Regenerated CT adjacent to root and newly formed bone showed significantly higher levels of OC, OPG, and OPG/RANKL in the P_11_-4-treated defects, suggesting a more favorable environment for osteogenesis; they also help explain the enhanced histological features of the newly formed bone. P_11_-4 is able to induce hydroxyapatite nucleation *de novo* (Kirkham et al., 2007), underpinning its use in enamel repair and regeneration (Schlee et al., 2013). Taken together with our own data, this SAP may be a promising osteoconductive scaffold.

It is well known that PR following PD is challenging due to the complexity of periodontal architecture (Lin et al., 2015). We have shown that the quality and organization of regenerated periodontal tissues appeared to be more favorable following the application of P_11_-4 compared with untreated defects. The orientation and organization of type I collagen appeared to be well developed 4 weeks after treatment with P_11_-4. In addition, AB showed enhanced trabecular configuration. This may be due to the unique features of the SAP including its resemblance to natural ECM which acts as a temporary template for the formative cells until replacement with newly formed ECM (Lin et al., 2015).

The normal histological structure of periodontium in the molar region in Sprague Dawley rats, is noted to show great resemblance to that of humans. The similarities include the structure and organization of junctional epithelium, periodontal collagen fibers and alveolar bone (Listgarten, 1975; Page and Schroeder, 1982; Ekuni et al., 2014). All study groups showed a degree of epithelial downgrowth when considering normal structure. This is known to be a common finding in periodontium being regenerated after loss (Usuda et al., 2004). However, our results showed more favorable results with P_11_-4 as shown by the significant decrease in epithelial downgrowth after 4 weeks in the P_11_-4 treated group. The orientation of regenerated oblique PDL fibers and the architecture of regenerated AB trabeculae in the P_11_-4 group was comparable to normal rat oblique PDL and AB, particularly after 4 weeks. On the other hand, the orientation of regenerated PDL fibers and the structure of regenerated AB was far less organized in the untreated group. The PDL length and AB height did not appear to return to their normal levels in any of the study groups. This may be due to the fact that the time points in our study were 2 and 4 weeks. Observation at a later time point may have given a larger window for structures to reach levels comparable to normal. However, when comparing the functional PDL length between both study groups, it was found to be significantly greater in the P_11_-4 treated group after 4 weeks. Also, on comparing AB height between the study groups at 4 weeks, it was found to be of higher values in the P_11_-4 group.

Our findings indicate that P_11_-4 treatment enhanced PR after 2 weeks and that this was further promoted 4 weeks post-surgery. Histomorphometry showed a significant increase in functional ligament length and a significant decrease in epithelial down-growth only after 4 weeks. In addition, although the relative AB height was less with P_11_-4 at 2 weeks, bone growth not only caught up with the untreated group but exceeded it after 4 weeks. The finding that OPG/RANKL ratios increased with time in P_11_-4 treated defects was particularly encouraging. This is consistent with previous investigations that found a significant effect for P_11_-4 after 4 weeks (Kirkham et al., 2007; Kyle et al., 2012).

The level of OC was greater in the P_11_-4 treated groups after 4 weeks. However, this increase was statistically non-significant. Evaluating the defects at a later time point, in addition to those included in the study, might have given more time for the regeneration to proceed and give us a deeper insight to the effects of P_11_-4 on periodontal tissue regeneration.

Although a degradation profile of P_11_-4 was not measured in this study, the findings of a previous study could offer an insight to this. P_11_-4 was proven to start resorbing after 14 days in the presence of dermal fibroblasts as opposed to the control group of P_11_-4 matrix without cells (Kyle et al., 2012). The authors concluded that the cells used the P_11_-4 matrix as a temporary scaffold over the 14 days to deposit ECM matrix. These findings show that the P_11_-4 when used *in vivo* may degrade at a rate similar to the rate of formation of new tissues.

Also another more recent study (Saha et al., 2019) investigated the effects of P_11_-4 in rat cranial defects. They showed that P_11_-4 degraded at a slower rate compared to P_11_-4 combined with HDPSCs. The authors also noted that defects filled with P_11_-4 alone demonstrated enhanced bone volume. It was concluded that this was more favorable for osteogenesis and bone remodeling as the ability of P_11_-4 nucleate hydroxyapatite crystals would be lost if p_11_-4 degraded at a quicker rate.

The acute defect model used here provides initial proof of concept and demonstrates the regenerative ability of P_11_-4. This was a surgically induced defect and not caused by chronic inflammation as is characteristic of human periodontal defects. Notwithstanding this, our study showed that P_11_-4 treatment clearly improved the outcome in terms of periodontal tissue regeneration compared with non-treated defects. In conclusion, P_11_-4 SAPs are promising candidates for the treatment of periodontal defects, used alone or as a scaffold combined with other components to enhance PR. Further investigations are required to determine its potential use in periodontal regenerative therapy.

## Data Availability Statement

The raw data supporting the conclusions of this article will be made available by the authors, without undue reservation.

## Ethics Statement

The animal study was reviewed and approved by Ethics Committee, Faculty of Dentistry, Mansoura University, Egypt.

## Author Contributions

BE-S, MEG, JK, and RE-G designed the study and experiments. BE-S and RD performed the experiments. BE-S, RD, RE-G, FI, and MEG contributed to the data analysis and interpretation. BE-S and RD drafted the manuscript. RE-G, FI, MEG, JK, and RE-Z critically revised the manuscript. All authors approved the final version of the manuscript.

## Conflict of Interest

JK is a named inventor of the self-assembling peptide technology used in this study. The remaining authors declare that the research was conducted in the absence of any commercial or financial relationships that could be construed as a potential conflict of interest.
