# Supplementary material for: An in-vivo Intraoral Defect Model for Assessing the Use of P11-4 Self-Assembling Peptide in Periodontal Regeneration
Source: Front Bioeng Biotechnol. 2020 Sep 23;8:559494. doi: 10.3389/fbioe.2020.559494 (PMC7550851; doi:10.3389/fbioe.2020.559494)
Supplement: Supplementary file 1 [file Table_1.DOCX]

**Appendix**

| **Appendix Table 1**: showing study design | | | | | | |
| --- | --- | --- | --- | --- | --- | --- |
|  |  |  |  |  |  |  |
| **Time Points** |  | **Rats** |  | **P_11_-4 defects** |  | **Untreated defects** |
|  |  |  |  |  |  |  |
| **Day 0** |  | **1 rat= 2 defects** |  | **1** |  | **1** |
|  |  |  |  |  |  |  |
| **2 Weeks** |  | **6 rats= 12 defects** |  | **6** |  | **6** |
|  |  |  |  |  |  |  |
| **4 Weeks** |  | **6 rats= 12 defects** |  | **6** |  | **6** |

| **Appendix Table 2**: showing different types of primary antibodies used in this study, their description, dilutions, cellular localization, Ag retrieval and detection kits. | | | | | | |
| --- | --- | --- | --- | --- | --- | --- |
| **Primary antibody** | **Abcam catalog number** | **Description** | **Dilution** | **Cellular localization** | **Ag Retrieval** | **2ry antibody kit** |
| Anti-PCNA antibody | ab29 | Mouse monoclonal | 1/500 | Nucleus | Sodium citrate 60°c  overnight | Abcam Mouse on Mouse Polymer IHC Kit |
| Anti-Osteocalcin antibody | ab13420 |  | 1/200 | Secreted | Sodium citrate  Microwave for 30 seconds then incubate at 37°c for 10 minutes  , finally allow to cool for 10 min at room temperature |  |
| Anti-Collagen I antibody | ab34710 | Rabbit polyclonal | 1/500 | Secreted>  extracellular space>  extracellular matrix |  | Novolink^TM^ Polymer Detection System (Leica Biosystems) |
| Anti-Osteoprotegerin antibody | ab73400 |  | 1/500 | Secreted |  |  |
| Anti-RANKL antibody | ab169966 |  | 1/500 | Cytoplasm, Secreted, cell membrane |  |  |
